# Dislodgement of Telescopic Nail from the Epiphysis: A Case Report with an Analysis of Probable Mechanism

**DOI:** 10.7759/cureus.7130

**Published:** 2020-02-28

**Authors:** Prateek Behera, John A Santoshi, Nikku M Geevarughese, Umesh Kumar K Meena, Rajkumar Selvanayagam

**Affiliations:** 1 Orthopaedics, All India Institute of Medical Sciences, Bhopal, IND; 2 Orthopaedics, Sawai Man Singh Medical College, Jaipur, IND

**Keywords:** telescopic nail, fassier-duval nail, complication, migration, pamidronate, sureshot, sofield and millar, osteogenesis imperfecta

## Abstract

Telescopic nails such as Fassier-Duval (FD) nails have become the standard treatment for stabilizing long bones and correcting deformities in osteogenesis imperfecta (OI). These nails do not require repeat surgery for their replacement when the bones outgrow them. However, they are not completely free from complications. The prohibitive costs of the original implants have led to design modifications being introduced in locally manufactured telescopic nails. While these low-cost devices work well in most cases, they can lead to complications resulting from their design flaws. We present here the complication observed in a locally manufactured telescopic nail with a design similar to the FD nail. The male component of the nail got dislodged from the distal tibial epiphysis, resulting in its proximal migration. We discuss the probable mechanism of this complication and propose possible design changes that can bring down the rates of such incidences.

## Introduction

Osteogenesis imperfecta (OI) is a rare heterogeneous group of inherited disorders characterized by brittle bones, frequent fractures, and skeletal deformities that affect an individual’s ability to walk [1]. The current medical treatment with intermittent doses of pamidronate has the potential to reduce pain and frequency of fractures, increase bone density, and improve function [2,3]. Surgical intervention aiming to brace the long bones (femur and tibia) from within (intramedullary) plays an important role in combined treatment concepts. Intramedullary devices help in decreasing the fracture rate and preventing deformities and have been postulated to encourage standing and walking [4]. In OI, tibial and femoral deformities result from traction forces of the muscles during bone growth following fractures. For the surgical treatment to be effective, the implant must act as a reinforcement device, transforming the bone segment into a more resistant structure. Primary indications for surgical corrections are recurrent fractures and unacceptable long bone deformity in patients who otherwise have the potential for standing and walking. Though surgery is ideal for children above three years of age, it must not be deferred in younger children having a high frequency of fractures [4].

Previously, fixation with plates was the usual practice for treating fractures in OI patients. However, experience has shown that they are poorly tolerated in structurally weak bones and secondary fractures frequently occur through screw holes and at the ends of the plates, as these are the areas of maximum stress concentration [3]. Intramedullary fixation of long bones has been an accepted method of treatment for OI patients since Sofield and Millar described their procedure of fragmentation, realignment, and intramedullary fixation of long bones [5]. However, a major problem associated with using a solid non-expandable intramedullary rod was the mandatory revision operation(s) after the implant became relatively short with bone growth. Bailey and Dubow introduced an elongating rod system, whereby a hollow sleeve and an internal obturator were engaged and anchored by T-pieces at the proximal and distal epiphyses of the long bones [6]. Over the years, experience with expansible nails has resulted in numerous design modifications being brought about to accommodate any reported complications.

Currently, Fassier Duval (FD) nail is one of the most commonly used telescopic nail systems [7]. Advantages of the FD nail over its predecessors include the ability to perform the surgery through a single entry point for insertion of both male and female rods and non-requirement of transverse stabilization, as it is fixed by its threaded ends into the epiphyses, and lesser number of nail replacement surgeries [8]. While the FD nail is an attractive option, its availability around the world is not uniform. Its prohibitive cost is among the factors affecting its availability, especially in developing countries. This has led to local manufacturers marketing it with design modifications. While this practice helps in making implants available to the masses at a fraction of the original cost, there may be associated problems that must be highlighted and brought to the notice of the scientific community so that adequate precautions can be taken when using them. Additionally, appropriate design changes may be suggested to improve the implants. We present the case of a patient in which using one such nails resulted in a complication that might have ramifications for the further management of the patient.

## Case presentation

**Figure 1:**
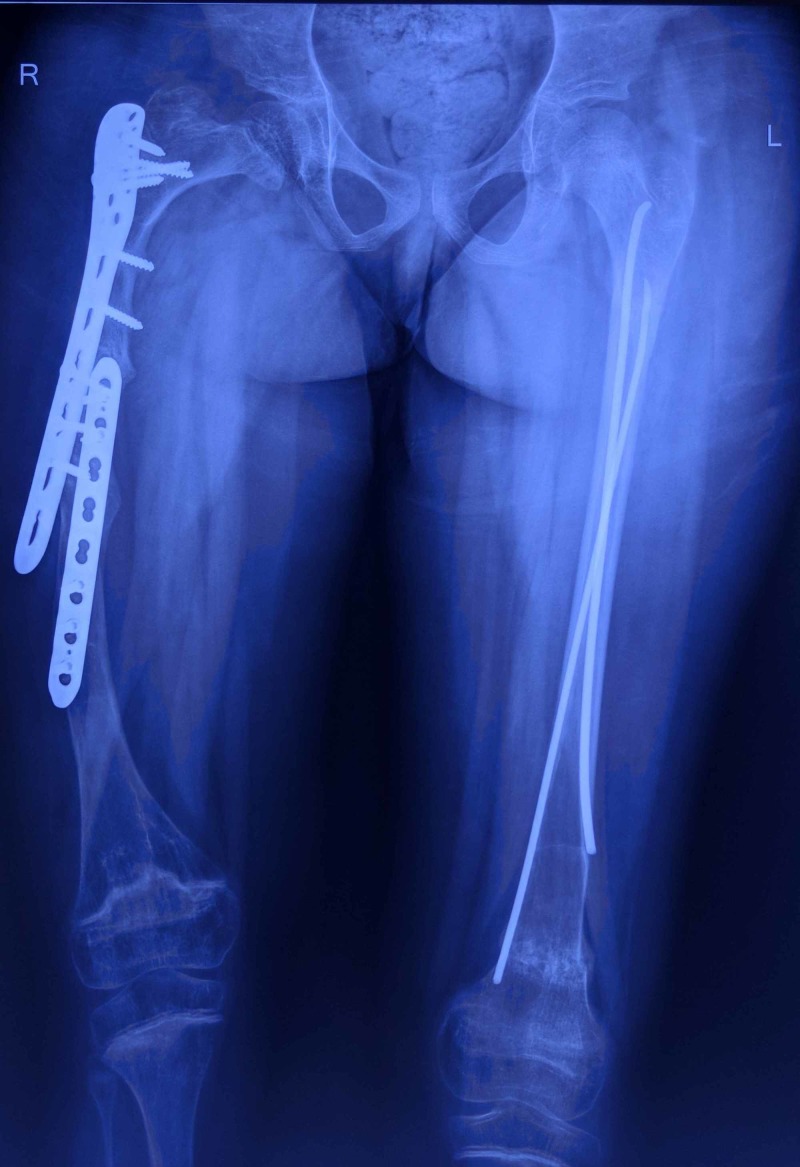
Anteroposterior radiograph of the patient at presentation showing LCP in situ on the right side and TENS on the left side LCP: locking compression plates; TENS: Titanium Elastic Nail System

The patient is a 10-year-old girl diagnosed with OI at the age of one. When she first consulted us, she had already undergone multiple surgeries for stabilization of femoral fractures with the use of devices like Rush nail, Küntscher nail, Titanium Elastic Nail System (TENS), and locked compression plates (LCP). At the time of presentation, she had groin-to-toe cast on her right lower limb apparently for the femoral fracture that she had sustained three months back. Radiographs of both the femur bones obtained after cast removal showed the presence of two LCPs on the right side and TENS nails on the left (Figure 1). 

**Figure 2:**
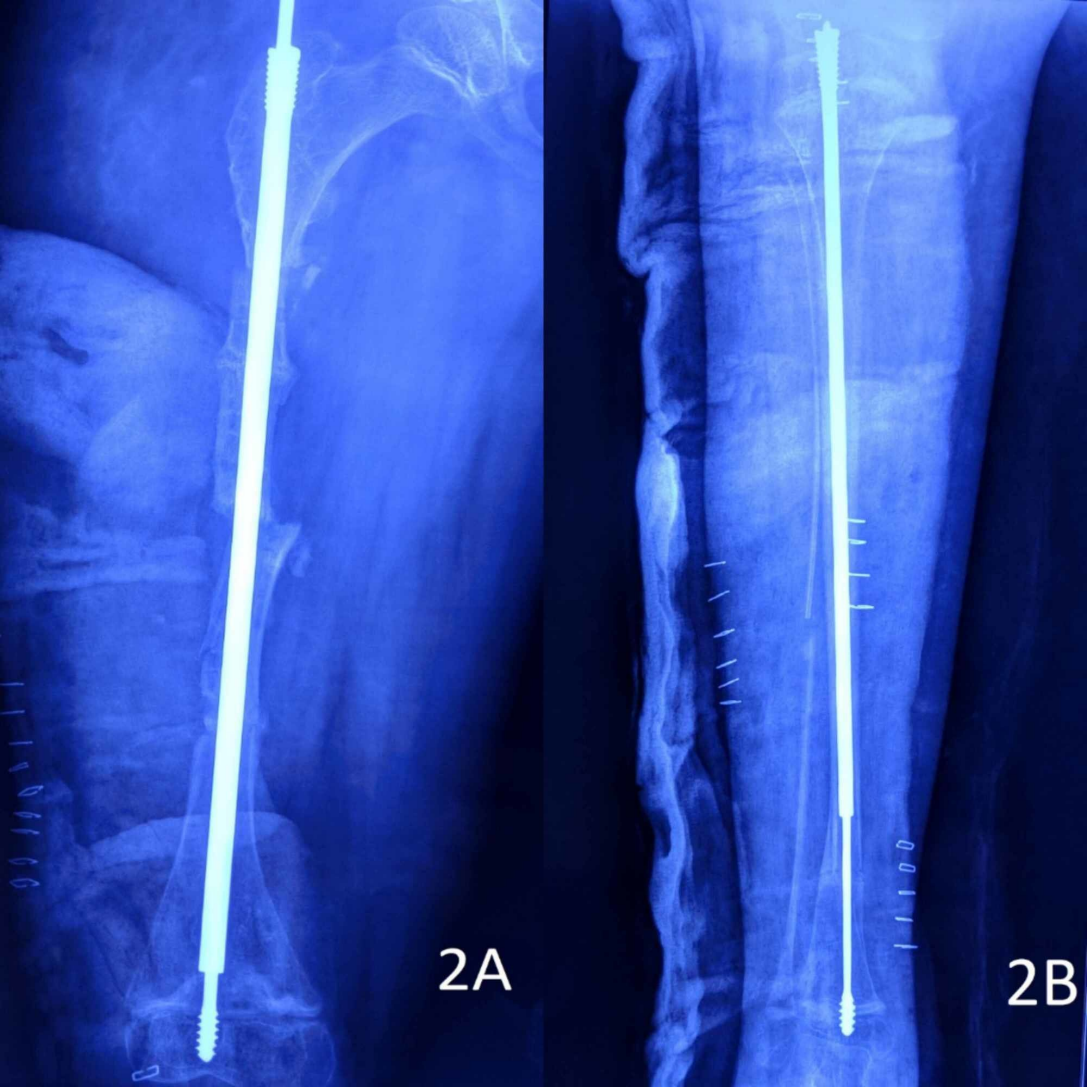
Immediate postoperative radiographs of the patient 2A: right femur; 2B: right tibia

She had been non-ambulatory for the past nine months. The parents were counselled about the benefits of pamidronate infusion reported in different studies and, with their consent, pamidronate infusion was started at 1 mg/kg body weight per day for three successive days. This three-day cycle was repeated every four months. Surgery was planned for deformity correction and telescopic nail insertion for both her lower limbs (both femur and tibia were planned to be stabilized). As the right side was more deformed, we decided to address it first. The technique described by Soffield and Millar was used [5]. After removing the LCP from the femur, entry for the nail was made using the awl provided with the instrumentation, under image intensifier guidance. Three open osteotomies for the femur and two percutaneous osteotomies for the tibia were performed. Both these bones were stabilized using locally manufactured telescopic nails (Universal Orthosystems, Vadodara, India) with a design similar to that of FD nail (Figure 2).

**Figure 3:**
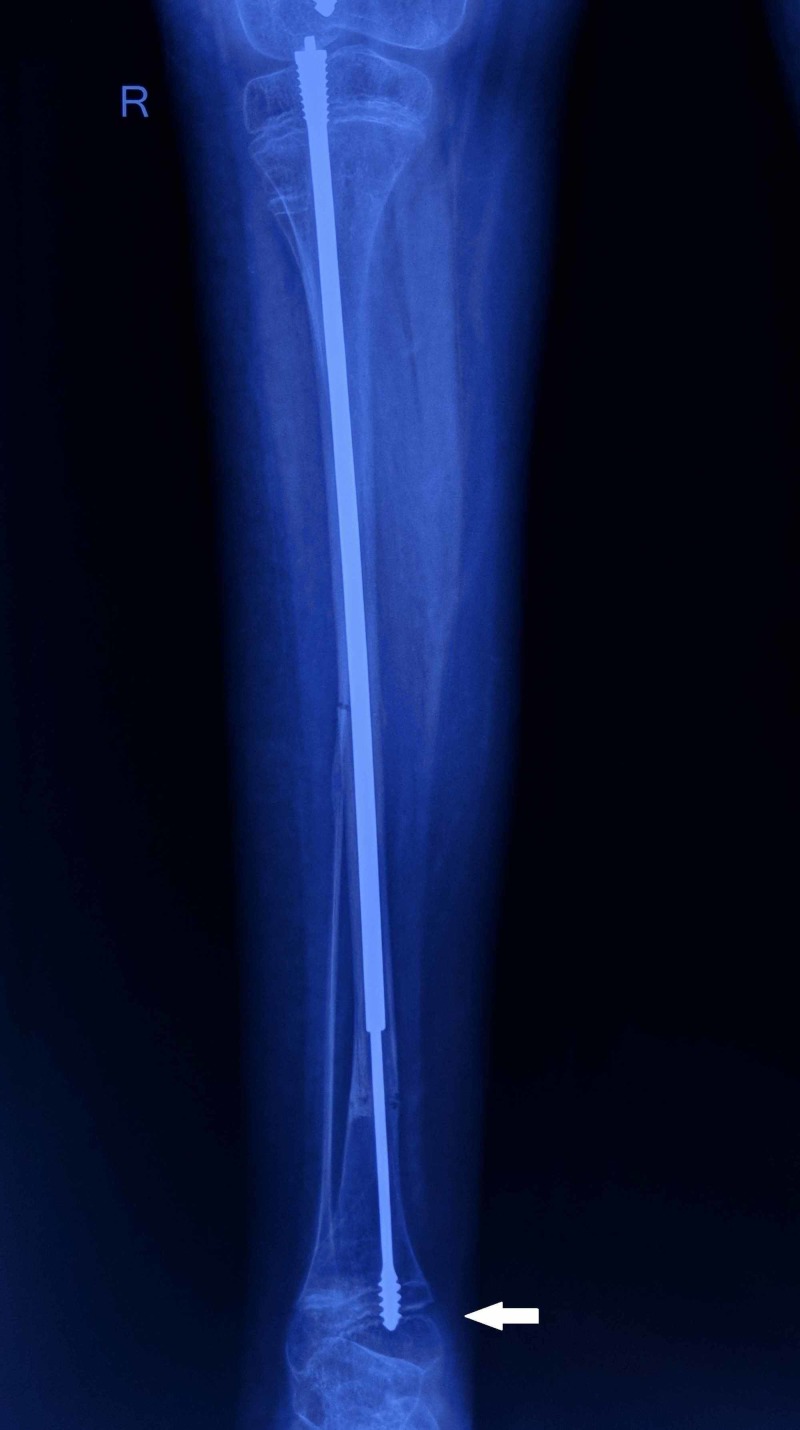
Follow-up radiograph of the right tibia The image shows the male component of the nail getting dislodged (white arrow) from the epiphysis

Following fixation, she was kept on an above-knee cast for six weeks. Knee range of motion (ROM) exercises were started after cast removal. Partial weight-bearing mobilization with a walker was initiated once radiological features of union were evident around the end of second month post-surgery. Nine months later, when she was planned for surgery on the left side, it was noted that threads of the male rod were getting dislodged from the right tibial epiphysis and the nail was starting to migrate proximally (Figure 3).

**Figure 4:**
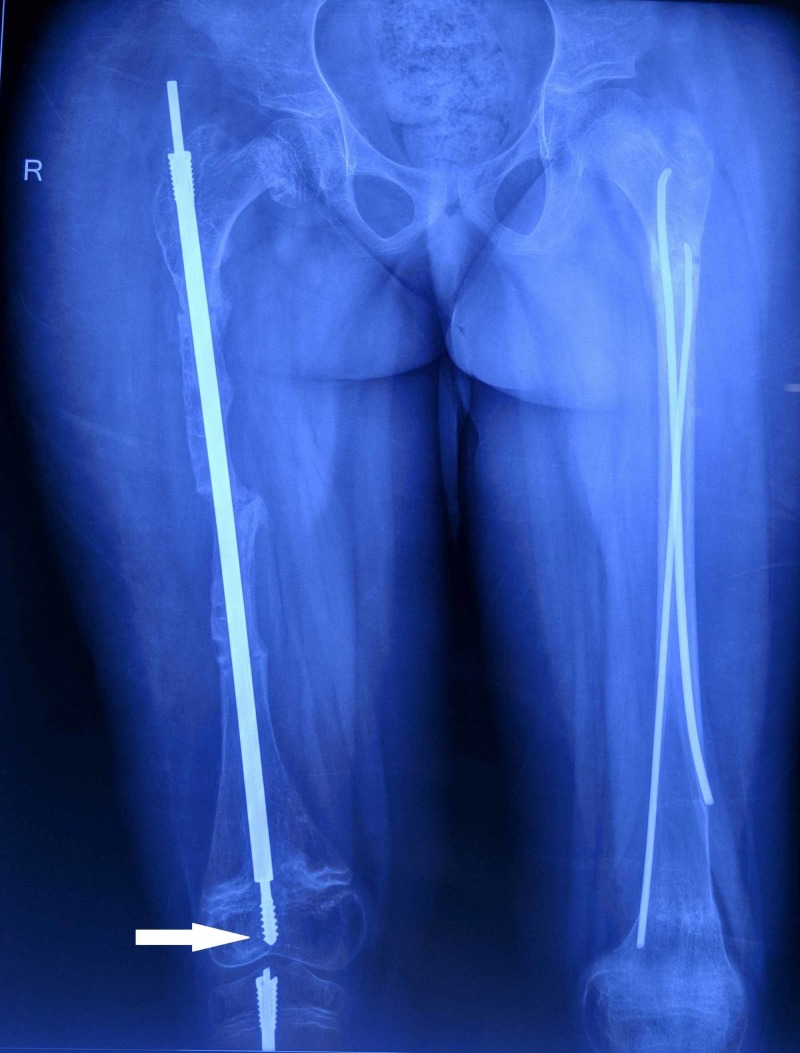
Follow-up radiograph of the right femur The image shows that the threads of the male rod are well embedded in the epiphysis (white arrow). The left femur has TENS in situ inserted at a different center TENS: Titanium Elastic Nail System

The threads of the tibial female component and those of the male and female components of the femur were in appropriate location (Figure 4).

**Figure 5:**
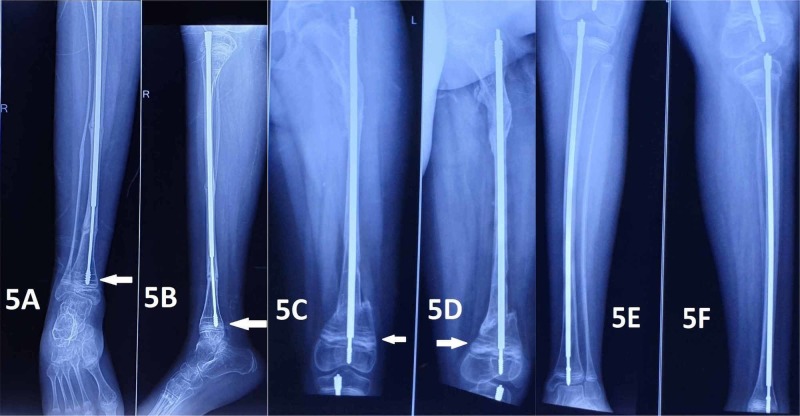
Radiographs obtained during the follow-up visit six months after the second surgery Figures 5A and 5B show the anteroposterior and lateral views of the right tibia with the male component dislodged from the tibial epiphysis (white arrow). Figures 5C and 5D show the anteroposterior and lateral views of the left femur, with the arrows showing the Park Harris lines. Figures 5E and 5F show the anteroposterior and lateral views of the left tibia where the nail was inserted without any osteotomy

We were faced with the challenge of either continuing with the same male rod in the tibia, exchanging it for another rod, or using a telescopic nail from a different manufacturer. It was decided not to intervene surgically as the provision for any other better designed telescopic rod system was unavailable. She underwent multiple osteotomies and telescopic nail stabilisation for the left femur and stabilization without any osteotomy for the left tibia. She has continued to receive pamidronate infusions at regular four-month intervals. Follow-up radiographs showed adequate lengthening of both femora and tibiae and she has been fracture-free till the last follow-up six months from the second surgery (Figure 5). She has been having regular four-month follow-ups to look for any complication arising from the proximal migration of the male rod and any angular deformity at the ankle. Her parents have been informed about the possibility of future surgical intervention in case it is deemed necessary to manage that complication.

## Discussion

Diamond nail, Hansen-Street nail, Ender nail, Schneider nails, Kuntscher nail, Rush rods, Steinmann pins, Kirchner wires, Bailey-Dubow, and Sheffield rods are few of the implants used over the years for the management of patients with OI [10]. Each of these devices has its fair share of complications. Non-elongating rods have higher reoperation and replacement rates than telescopic rods. Although elongating rods have the benefit of expanding as the bone grows, non-elongating rods are still preferred when treating either the too young, whose bones are too small to support telescopic rods, or those nearing skeletal maturity whose bones are no longer expected to grow [11]. Several single proximal-entry telescopic nails have been developed after the Bailey-Dubow and Sheffield rod, such as the Santa Casa telescopic intramedullary rod, Dyna-Locking Telescopic Rod, and the Fassier-Duval (FD) nail [7,12]. FD nails have widespread acceptance in several pediatric orthopedic centers around the world. Telescopic rods are known to have several complications like disassembly of the telescopic system, rod bending or breakage, rod migration, migration of components of its locking system, joint or skin penetration, growth stimulation as well as the arrest of growth, non-elongation, fractures, non or delayed union, and infection [4]. Failure to telescope adequately has been reported by surgeons using the FD nails. In regular practice, the most likely reason for the dislodgement of threads of the male rod is due to improper cutting and filing of the proximal-most end of the male rod. However, in the present case, the male rod was sharply cut and appropriately filed.

**Figure 6:**
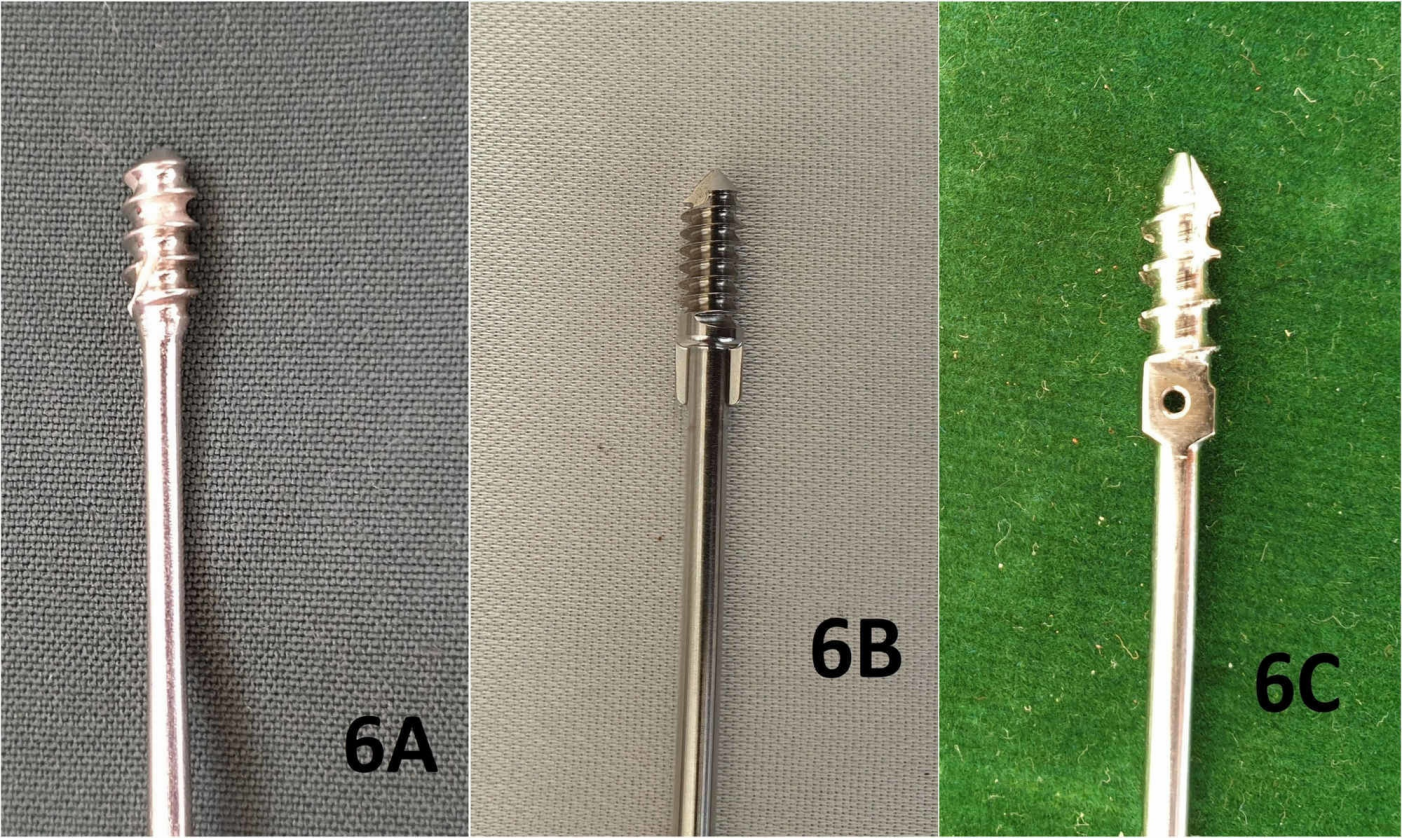
Comparison of the designs of three different Fassier-Duval nails Figures 6A, 6B, and 6C show the profile and features of the tips of the male rods manufactured by Universal Orthosystems, Pega Medical, and Uma Surgicals respectively

Based on the study of the implant designs on offer by different manufacturers, the proximal dislodgement of the threads of the male rod from the distal tibial epiphysis in the above-described case can primarily be attributed to the persistence of the threads in the physis and metaphysis and inadequate purchase in the distal tibial epiphysis. If one analyzes the design of the male rod used in this case from the local manufacturer (Universal Orthosystems, Vadodara; Figure 6 A), one can find that there is a higher pitch of the epiphyseal threads as compared to the FD nail manufactured by Pega Medical (Quebec, Canada) (Figure 6B). The male rod from another local manufacturer (Uma Surgicals, Mumbai; Figure 6C) has a higher pitch of the threads too, and it has an additional hole for accommodating a K-wire. Theoretically, a K-wire inserted through this hole gives the surgeon an option of improving the stability of the construct. However, placing this K-wire is extremely challenging as the hole is very small and surgeons may abandon this step. On analyzing the male rods of both the locally manufactured FD nails, it is evident that the higher pitch of the threads would practically result in the placement of only one or one-and-half threads in the epiphysis. Most of the threads would be in the physis and the metaphysis. In concurrence with previous studies, we considered that revision in the given case would demand a higher dimension of surgery and, since a better implant than the currently used one was unavailable, we decided against intervening at present [13,14].

As we were not able to find a similarly reported case, we believe that raising awareness among the surgeons using these nails is a must so that they are able to monitor patients adequately for this complication. It is unlikely that these nails manufactured by local manufacturers will be avoided by surgeons in view of this complication. But steps can be taken for designing better nails to prevent such a complication. Birke et al. and Mansour et al. have offered a few suggestions to improve the design of the FD nails [15,16]. Additionally, the implant design of the femoral and tibial components could be made different from one another. The present practice of using implants with similar designs for both femur and tibia should be replaced with bone-specific designs and sizes. Tibial male rods with different combinations of the pitch of threads and length of the threaded part should be made available in the set. That is, for a given length of the threaded part, rods with different thread pitch should be available for the surgeon to choose from, who can then ensure that at least two to three full threads are fully buried in the epiphysis. The size of the hole for K-wire placement could be made larger and the use of technology like that of Trigen Sureshot (Smith & Nephew, London, UK) should be incorporated for easy placement of K-wire [17]. The use of flanges to improve distal fixation like arthroplasty systems could be evaluated [18]. Additionally, it will be helpful if the cost of patented implants is lowered for developing countries or if the parent company licenses a local manufacturer for manufacturing its implants at a local level in the exact design as the original one. It would be beneficial to the scientific community if reports of any similar complication with telescopic nails of other manufacturers and designs are also brought out.

## Conclusions

Telescopic nails such as FD nails have vastly improved the lives of OI patients by allowing them greater mobility with fewer surgeries. Pamidronate infusion also helps in the management of such patients by preventing refractures. Facsimile nails of the original FD nails work well in most cases, but they can have design flaws resulting in complications. Orthopedic surgeons using such nails should be aware of these flaws and should be prepared to deal with any future complications arising due to them.
